# Partial duplication of the *PRLR *and *SPEF2 *genes at the late feathering locus in chicken

**DOI:** 10.1186/1471-2164-9-391

**Published:** 2008-08-20

**Authors:** Martin G Elferink, Amélie AA Vallée, Annemieke P Jungerius, Richard PMA Crooijmans, Martien AM Groenen

**Affiliations:** 1 Animal Breeding and Genomics Centre, Wageningen University and Research Centre, P.O. Box 338, 6700 AH Wageningen, The Netherlands; 2 Breeding Research and Technology Centre; Hendrix Genetics, P.O. Box 30, 5830 AE Boxmeer, The Netherlands

## Abstract

**Background:**

One of the loci responsible for feather development in chickens is K. The K allele is partially dominant to the k+ allele and causes a retard in the emergence of flight feathers at hatch. The K locus is sex linked and located on the Z chromosome. Therefore, the locus can be utilized to produce phenotypes that identify the sexes of chicks at hatch. Previous studies on the organization of the K allele concluded the integration of endogenous retrovirus 21 (ev21) into one of two large homologous segments located on the Z chromosome of late feathering chickens. In this study, a detailed molecular analysis of the K locus and a DNA test to distinguish between homozygous and heterozygous late feathering males are presented.

**Results:**

The K locus was investigated with quantitative PCR by examining copy number variations in a total of fourteen markers surrounding the ev21 integration site. The results showed a duplication at the K allele and sequence analysis of the breakpoint junction indicated a tandem duplication of 176,324 basepairs. The tandem duplication of this region results in the partial duplication of two genes; the prolactin receptor and the gene encoding sperm flagellar protein 2. Sequence analysis revealed that the duplication is similar in Broiler and White Leghorn. In addition, twelve late feathering animals, including Broiler, White Leghorn, and Brown Layer lines, contained a 78 bp breakpoint junction fragment, indicating that the duplication is similar in all breeds. The breakpoint junction was used to develop a TaqMan-based quantitative PCR test to allow distinction between homozygous and heterozygous late feathering males. In total, 85.3% of the animals tested were correctly assigned, 14.7% were unassigned and no animals were incorrectly assigned.

**Conclusion:**

The detailed molecular analysis presented in this study revealed the presence of a tandem duplication in the K allele. The duplication resulted in the partial duplication of two genes; the prolactin receptor and the gene encoding sperm flagellar protein 2. Furthermore, a DNA test was developed to distinguish between homozygous and heterozygous late feathering males.

## Background

One of the loci responsible for feather development in chickens was described by Serebrovsky in 1922 [1] and is designated by the symbol K, standing for 'kürzer flügel' (short wing) [2]. The K allele is associated with the late feathering phenotype (LF) that causes a retard in the emergence of primary and secondary flight feathers. The k+ allele is associated with the early feathering phenotype (EF), resulting in the earliest emergence of feathers. The K allele appears to be incompletely dominant to k+, resulting in phenotypes with different intensities due to a dosage effect of the locus [3]. For more detailed information about the feathering loci, see the extensive review by Chambers et al. [4].

In birds, sex is determined by two chromosomes, Z and W. Males are homozygous ZZ and females are hemizygous ZW. The K locus is located on the Z chromosome and can be utilized to produce phenotypes that distinguish between the sexes of chicks at hatching, but also at the embryonic stage [5,6]. This method of sexing based on differences in the rate of feather growth provides a convenient and inexpensive approach.

Although the LF phenotype facilitates the sexing of chicks, the K allele is also associated with a reduction in egg production, an increase in infection by lymphoid leucosis virus [7], and an increase in the mortality rate [8]. These negative side effects may be caused by the presence of the endogenous retrovirus 21 (ev21) [8]. Concordance between expression of ev21 and the LF phenotype indicated a linkage of less than 0.3 cM between K and the ev21 locus [9,10]. The ev21 locus consists of an integration site that can be occupied (ev21+) or unoccupied (ev21-). EF animals were found to have only one unoccupied site per Z chromosome; whereas, LF animals have at least one Z chromosome with an unoccupied and an occupied site [11]. A study on the organization of the K allele concluded the integration of ev21 into one of two large homologous segments located on the Z chromosome of LF chickens [12]. EF revertants carrying an occupied site have been observed; therefore, it was concluded that ev21 itself could not be the sole cause of the LF phenotype [13].

Several tests have been developed to identify the EF and LF alleles [12,14,15]. These tests focused on the presence of the occupied and unoccupied site in the genome. Unfortunately, even if these methods are fully informative when applied to females, they do not allow for differentiation between homozygous and heterozygous males. Furthermore, the existence of ev21-positive EF animals will give false-positive results with these tests.

In this study we present a detailed molecular analysis of the K locus and develop a DNA test to distinguish between homozygous and heterozygous late feathering males.

## Results

### Molecular analysis of the K locus

A quantitative PCR (qPCR) approach, as described by Weksberg et al. [16], was used to investigate the K locus. Copy number variation was determined at fourteen markers (STS_1-STS_14) designed to surround the ev21 integration site (Table 1). In two chickens, the most likely location of the duplicated block was mapped between markers STS_6 and STS_13 (Table 2). Marker STS_5 and marker STS_6 gave ambiguous results (Table 2).

**Table 1:** STS markers used in the molecular analysis of the K locus.

**Marker Name**	**Location^1 ^(bp)**	**Position**	**Sequence**	**Length (bp)**
STS_0	80092619^2^	Forward	CACACAGAAGACGGTGGATG	170
	80092788^2^	Reverse	TGGCTCCTACCTCCTGACAC	
STS_1	9764119	Forward	GAAGGAGAGCCTGTTTGCTG	207
	9764325	Reverse	CTTGTGGTGGTGAAGTGGTG	
STS_2	9862778	Forward	AAGTGGGACAACGGAAAGAC	345
	9863122	Reverse	AGGTCAAAGAAGGCACAAGG	
STS_3	9913200	Forward	AGCCAGAAACAAAAGCCAAA	148
	9913347	Reverse	TCAGCCTCGACACAGAAAAA	
STS_4	9933229	Forward	AGTGTCAGTGTGCCTCTTGG	170
	9933398	Reverse	CACGGCATTTATGAGATTGG	
STS_5	9950543	Forward	AATCAGAGTTGCAGGGGTTG	135
	9950677	Reverse	TTGACTGGGGCTCAATAAGG	
STS_6	9960545	Forward	TCTCCCTCCCTGTCTTCTCA	215
	9960759	Reverse	TGGCCTTGAAAATCCTCTTG	
STS_7	9973781	Forward	TAGCAGACAAGGGCATTCAG	198
	9973584	Reverse	GCATTTGTAGGGCTGGATTTG	
STS_8	9996871	Forward	ACCAAAGCGTCCAAAATGTC	198
	9997068	Reverse	TACCAGGGGAGAGCATGAAG	
STS_9	10038160	Forward	AAATAGGCACGAGGGAAGC	176
	10037985	Reverse	AACCATCAAGACTGGCTCAAC	
STS_10	10078039	Forward	GCCCTCTAAGTGCCTGACTG	182
	10078220	Reverse	TTTCATGCGTAGGAGCTGTG	
STS_11	10106858	Forward	CACTTCCAGGGTTGGTGACT	343
	10107200	Reverse	GAGGGCATCCATCACATCTC	
STS_12	10135701	Forward	TGGAGCTGAGGAAAGAATCC	105
	10135805	Reverse	TGCTTGCAGGTTTGAGTGTC	
STS_13	10168014	Forward	TCCACTTGTCATGCACTTCC	179
	10168192	Reverse	AAGTTCCCCAAAAATACTGCTG	
STS_14	10181226	Forward	TGTGAGCAATTCCATTCTGG	216
	10181441	Reverse	TTGGTTCAGTTTGGTCATCG	
STS_Junction	10141819	Forward	CTGAGAGTGTTGTCCCAGCA	1432^3^
	9966922	Reverse	TGTTGAGTGCTCTTGGTTGC	
STS_Control	9899810	Forward	ACGCTGGCTTTCCCAACAG	70
	9899879	Reverse	AGACTGCCACATACCAGAAGCA	
STS_Break	10142644	Forward	ACAAGTGTCAGACTAGGGCTAGCA	78^3^
	9966396	Reverse	TGAAACCATCCCTGGAGAGATG	
STS_5block	9965590	Forward	ACCATTTCCACATTCCCTTCT	1333
	9966922	Reverse	TGTTGAGTGCTCTTGGTTGC	
STS_3block	10141819	Forward	CTGAGAGTGTTGTCCCAGCA	1289
	10143107	Reverse	CGGGCCATTATTTCATTTTG	

^1^Genomic location on the Z-chromosome in basepairs (WASHUC2 assembly), ^2^Marker STS_0 is located on chromosome 1, ^3^in late feathering animals only.

**Table 2:** The ΔKCt values for the STS markers in two chickens.

**Breed^1^**	**Sex**	**STS_1**	**STS_2**	**STS_3**	**STS_4**	**STS_5**	**STS_6**	**STS_7**
BR	Male	0.05	0.20	0.21	0.11	*0.40*	0.20	**1.17**
WL	Male	-0.03	-0.04	0.33	-0.04	0.01	*0.38*	**1.24**
								

**Breed^1^**	**Sex**	**STS_8**	**STS_9**	**STS_10**	**STS_11**	**STS_12**	**STS_13**	**STS_14**

BR	Male	**1.49**	**1.52**	**1.71**	**1.19**	**1.36**	0.29	0.14
WL	Male	**1.11**	**1.21**	**1.62**	**1.13**	**1.23**	0.13	0.15

^1 ^BR: Broiler, WL: White Leghorn. Normal font indicates a ΔKCt ≤ 0.35. An italic font indicates a ΔKCt >0.35 and <0.65. A bold font indicates a ΔKCt ≥ 0.65.

**Figure 1:**
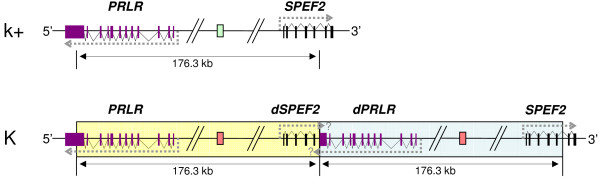
**The organization of the k+ and K alleles**. The k+ allele contains two genes; *PRLR *(purple exons) and *SPEF2 *(black exons). The K allele contains the original genes and the two partial duplicate genes, *dPRLR *and *SPEF2*. The green box indicates the unoccupied ev21 integration site. One of the red boxes indicates an unoccupied and the other an occupied ev21 integration site. The large yellow and blue boxes indicate the 176.3 kb duplicated block. The grey arrows indicate transcriptional start and stop. The question mark indicates a transcript of unknown length.

To determine the size and orientation of the duplicated block, forward and reverse primers were designed for both ends (between marker STS_6 and STS_7, and between markers STS_13 and STS_14). A 1238 bp product was obtained spanning the breakpoint junction (marker STS_junction) in two late feathering males. With this marker, no PCR product was obtained from the DNA of the two EF birds. Sequence analysis of the PCR product obtained from the two LF males provided the exact breaking point. Based on the WASHUC2 assembly, the total length of the tandem duplication is 176,324 bp (GGAZ 9,966,364–10,142,688 bp). The tandem duplication of this region results in the partial duplication of two genes: the prolactin receptor (*PRLR*) and the gene encoding sperm flagellar protein 2 (*SPEF2*, also known as *KPL2*). The duplicated block included exons 1 to 11 and 558 bp of exon 12 of *PRLR*, and exons 1 to 5 of *SPEF2 *(Figure 1). No differences in the nucleotide sequences of the breakpoint junction fragments were observed between the Broiler and White Leghorn animals.

To validate the duplication, a PCR reaction was performed with a new marker spanning the breakpoint junction (STS_break). The experiment was performed on twelve EF and twelve LF animals from eight different lines. No band was observed for the EF animals; whereas, all LF animals showed the 78 bp band corresponding to the breakpoint junction.

To obtain information about possible aberrations at the ends of the duplication, both regions were sequenced (markers STS_5block and STS_3block). No sequence differences were found between the LF and wildtype (EF) animals.

### DNA test to distinguish between homozygous and heterozygous late feathering males

The breakpoint junction was used to develop a TaqMan-based DNA test that can distinguish between homozygous and heterozygous LF males (further referred to as the TaqMan K test). Two TaqMan markers were used: one outside the duplicated block (marker STS_control) was used as a control and one spanning the breakpoint junction (marker STS_break) was used for investigating the duplication (Table 1). Two minor groove binding (MGB)-probes were designed for these markers, the MGB-control probe (TCTGTCCAAACATTTATTTG) was labeled with the fluorescent dye VIC and used for the control marker STS_control, and the MGB-Break probe (CCCTTAAATGCCTTGCTT) was labeled with the fluorescent dye FAM and used for the breakpoint junction marker STS_break. To validate the TaqMan K test, 25 animals were tested in duplicate. Eight randomly selected reference animals (four K/K and four K/k+) were used to determine the range of K/K and K/k+ animals in each experiment (Table 3). Seventeen animals with known genotypes were used to validate the ranges (Table 4). In the first experiment, an animal was considered K/K if the ΔCt was between 0.68 and 1.43 or K/k+ if the ΔCt was between 1.75 and 2.50. For the second experiment, the range of ΔCt for K/K was between 0.63 and 1.24 and between 1.50 and 2.10 for K/k+. Based on these calculations, 94.1% of the animals in the first experiment were within the ranges of their known genotype (correctly assigned), and 5.9% were outside either range (unassigned). No animals were false positive (incorrectly assigned). In the second experiment, 76.5% of the animals were correctly assigned, 23.5% were unassigned and no animals were incorrectly assigned. In total, 29 of the 34 validation animals (85.3%) were correctly assigned, 5 animals (14.7%) were unassigned and no animals were incorrectly assigned.

**Table 3:** The TaqMan-based DNA test for the K allele on reference animals.

Animal ID	Genotype	Experiment 1 ΔCt	Experiment 2 ΔCt
6333	K/K	0.92	0.79
4148	K/K	1.14	0.77
4384	K/K	1.16	1.13
6323	K/K	1.00	1.05
			
949	K/k+	2.15	1.76
6182	K/k+	2.09	1.62
2636	K/k+	1.90	1.66
947	K/k+	2.38	2.14
			
Average	K/K	1.06	2.13
	K/k+	0.94	1.80

**Table 4:** The TaqMan-based DNA test for the K allele validated on late feathering K/K and K/k+ animals.

Animal ID	Known Genotype	Experiment 1 ΔCt	Experiment 1 Genotype	Experiment 2 ΔCt	Experiment 2 Genotype	
2864	K/k+	0.76	K/k+	0.64	K/k+	
B2L4	K/k+	0.68	K/k+	0.49	Unassigned	
942	K/k+	0.90	K/k+	1.01	K/k+	
2855	K/k+	0.98	K/k+	0.87	K/k+	
4117	K/k+	1.10	K/k+	0.40	Unassigned	
4118	K/k+	0.98	K/k+	0.83	K/k+	
4332	K/k+	1.31	K/k+	1.14	K/k+	
6388	K/k+	1.06	K/k+	0.77	K/k+	
6324	K/k+	1.12	K/k+	0.91	K/k+	
6130	K/K	2.44	K/K	1.84	K/K	
6297	K/K	2.09	K/K	1.40	Unassigned	
952	K/K	2.09	K/K	1.74	K/K	
1030	K/K	1.83	K/K	1.90	K/K	
2849	K/K	2.26	K/K	1.64	K/K	
6187	K/K	2.10	K/K	1.85	K/K	
6242	K/K	1.73	unassigned	1.50	K/K	
6172	K/K	1.93	K/K	1.47	Unassigned	

	Experiment 1		Experiment 2		Total	
			
	Animals (n = 17)	%	Animals (n = 17)	%	Animals (n = 34)	%

Correct	16	94.1	13	76.5	29	85.3
Incorrect	0	0.0	0	0.0	0	0.0
Unassigned	1	5.9	4	23.5	5	14.7

The seventeen animals were validated based on the ranges found for K/K and K/k+. For experiment 1, the ΔCt range for K/K was 0.68–1.43 and for K/k+ 1.75–2.50. For experiment 2, the ΔCt range for K/K was 0.63–1.24 and for K/k+ 1.50–2.10.

## Discussion

The detailed molecular analysis presented in this study confirmed the presence of the duplication first described by Iraqi and Smith [12]. The total size of the tandem duplication is 176,324 bp, which is in agreement with the estimated 180 kb [12]. Sequence analysis found that the duplication is similar in both Broiler and White Leghorn lines, and all 12 LF animals showed the 78 bp breakpoint junction fragment (marker STS_break in the current study) indicating that the duplication is similar in all animals. This suggests that the duplication was of the same origin for all three breeds, and that the duplication most likely occurred in a common ancestor. On the other hand, since the K allele is extensively used by breeders, it is also likely that this particular allele was introduced into all three breeds.

In theory, the values of unaffected and duplicated markers should be equal to 0 or 1, respectively, in the qPCR experiments. However, ΔKCt varied from -0.04 to 1.71, and markers STS_5 and STS_6 had ambiguous results (Table 2). This variation is likely to be due to biological variations and the fact that the experiment was only performed once with two animals.

The observed duplication could be the result of an unequal recombination event in the Z chromosome. However, no apparent sequence homologies are found in the two areas involved in the duplication. Therefore, the unequal recombination event is not supported by our data, although a nonhomologous recombination event can not be excluded. Alternatively, integration of ev21 resulted in the duplication at the K locus. This raises the possibility of additional duplications at other locations in the chicken genome, which contains approximately 12,000 copies of long terminal repeats (1.3%) belonging to the vertebrate-specific class of retroviruses [17]. However, the actual ends of the duplicated block are located approximately 70 kb upstream and 103 kb downstream of the ev21 integration site, making this possibility less likely.

**Figure 2:**
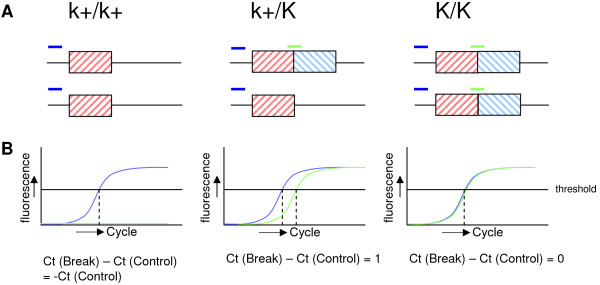
**Difference in the Ct values of homozygous early feathering (EF), heterozygous late feathering (LF), and homozygous LF animals**. **A) **Comparison of K locus components on the Z chromosomes of different genotypes. The red striped box and blue striped box indicate the duplicated blocks of genetic sequence. The dark blue line is marker STS_control and the green line is marker STS_break. **B) **The theoretical curves of the qPCR. In k+/k+ animals the difference between Ct (Break) and Ct (Control) will be -Ct (Control). For k+/K animals the theoretical difference will be 1 Cycle. For K/K animals the difference will be 0.

A PCR amplicon spanning the breakpoint junction is sufficient for distinguishing LF birds from EF birds. In males however, the challenge was to be able to differentiate between LF homozygous (K/K) and LF heterozygous (K/k+) animals. In this study, we found that the duplicated block is specific for the K allele and it was used to develop a DNA test based on the breakpoint junction. Since the PCR reactions in the TaqMan K test are performed in a multiplex, the concentration of DNA, theoretically, has no influence on the ΔCt. This contributes to the robustness of the test since variations in the concentration of DNA between and within test and control animals does not have an influence on the results. The ΔCt value gives an indication of the haplotype of an animal. In theory, when ΔCt is equal to 1, the animal is heterozygous, and when ΔCt is equal to 0, the animal is homozygous (Figure 2). In the TaqMan K test experiments, the homozygous reference animals had an average ΔCt of 1.06 and 0.94, and the heterozygous reference animals had an average ΔCt of 2.13 and 1.80 (Table 3). This difference from the theoretical value was most likely caused by the different efficiencies of the markers.

The aim was to develop a highly reliable test that is convenient for intensive use. The reliability of the test was defined by the percentage of correctly and incorrectly assigned animals. The TaqMan K test was validated using eight reference and seventeen validation animals in duplicate. Of the validation animals tested, 85.3% were identified correctly, 14.7% were unassigned, and no animals were incorrectly assigned (Table 4). Based on the literature, no previous test has been capable of identifying LF homozygous and LF heterozygous males with this level of reliability.

Although the LF phenotype facilitates the sexing of chicks at hatching, expression of ev21 is associated with the negative side effects of the K allele [7,8]. The establishment of a line where late-feathering is not associated with decreased egg production and tolerance to exogenous avian leucosis virus infection would be of prime commercial interest. Obviously, the search for the K allele lacking the occupied site is an effective approach. This search for revertants and the establishment of a line can be done by combining the TaqMan K test and the ev21 test proposed by Tixier-Boichard [15].

The observed duplication resulted in the partial duplication of two genes: *PRLR *and *SPEF2 *(Figure 1). The genes are oriented in opposite directions; therefore, the duplication event does not result in a fusion gene. However, alternative transcripts of the partially duplicated genes may be found. Interestingly, the transcript of both partially duplicated genes could contain the antisense sequence of the other gene, which could lead to RNA interference and influence the translation of both the duplicated and original genes.

The membrane-bound PRLR is closely related to the growth hormone receptor and is a member of the cytokine receptor family [18]. The pituitary hormone, prolactin (PRL), is a ligand of PRLR. More than 300 separate biological activities have been attributed to PRL: reproduction, endocrine signaling and metabolism, control of water and electrolyte balance, growth and development, neurotransmission and behavior, and immunoregulation and protection [19]. More detailed functions of PRL include involvement in the control of seasonal pelage cycles [20-22], egg production [23], and the induction of molting [24]. Furthermore, PRL is involved in the immune system [25], autoimmune diseases, and the growth of different forms of cancer [18].

In *PRLR *(-/-) knockout studies on mice, the normal progression of hair replacement and follicle development have been observed [26]. These knockout mice showed a change in the timing of hair replacement and molting, and both phenotypes are advanced compared to the wild type. It was concluded that knocking out *PRLR *shortens the telogen phase of the hair cycle and advances the anagen phase of hair follicles [26,27]. Therefore, it can be suggested that PRLR plays an inhibitory role in follicle activation.

The relatively unknown protein, SPEF2, is believed to play an important role in the differentiation of axoneme-containing cells [28]. Truncation of the SPEF2 protein results in immotile short-tail sperm in pigs [29]. Due to the presence of an ATP/GTP binding site and a proline rich domain, it was suggested that SPEF2 might be involved in signal transmission [28].

The actual cause of delayed feathering is still unknown. It can be speculated that *PRLR*, due to its inhibitory role in follicle activation, is the major candidate gene involved in this delay. *SPEF2 *may be involved in the transmission of signals in the feather growth pathway. Further research is needed to confirm the involvement of these genes, which could focus on 1) the truncated proteins formed by *PRLR *or *SPEF2 *as a result of the partial duplication, 2) the transcripts of the partially duplicated genes and their influence on the expression and translation of the two original genes, and 3) the expression of (partially duplicated) *PRLR *and *SPEF2 *that may have changed due to the rearrangement, duplication, or deletion of regulatory elements.

Although it has been extensively described that ev21 causes the negative side effects of the K allele, the findings of this study might also indicate involvement of *PRLR*. As described above, prolactin and its receptor are involved in the growth of different forms of cancer [18], egg production [23], and in the immune system [25]. Because the negative side effects of the K allele include an increase in infection by lymphoid leucosis virus, an increased mortality, and a reduction in egg production, it can be speculated that the partial duplication, altered expression, or altered translation of *PRLR *might also be involved in the negative side effects. If the partial duplication of *PRLR *is responsible for the delay in feather growth, and contributes to the negative side effects, it will not be possible to separate the advantageous and disadvantageous effects of the K allele.

## Conclusion

The detailed molecular analysis presented in this study indicates the presence of a 176,324 bp tandem duplication in the K allele. An identical duplicated block is found in Broiler, White Leghorn, and Brown Layer lines. The duplication results in the partial duplication of two genes: *PRLR *and *SPEF2*. Due to its inhibitory role in follicle activation, *PRLR *is the most likely candidate gene involved in the delay of feather growth. However, *SPEF2 *may be involved in the transmission of signals in the feather growth pathway.

In addition to the characterization of the K locus, a DNA test was developed to distinguish between homozygous and heterozygous late feathering males. The percentage of animals correctly assigned was 85.3%, while 14.7% were unassigned. No animals were incorrectly assigned. To date, this is the most reliable and robust DNA test developed to differentiate between LF homozygous and LF heterozygous males, and would be indispensable in decreasing errors generated by crossing animals with incorrect genotypes.

## Methods

### DNA collection

Chicken genomic DNA was extracted from the blood of EF and LF animals provided by Hendrix Genetics (the Netherlands) using the Puregene DNA purification blood kit (Gentra System, USA). DNA concentration and quality were measured using the Nanodrop ND-1000 spectrophotometer. In total, 14 homozygous EF males (k+/k+), 23 homozygous LF males (K/K), three LF females (K/W), and 12 heterozygous LF males (K/k+) from three different lines (Broiler, White Leghorn, and Brown Layer) were used. The genotypes were determined by examining the feathering phenotypes of their offspring.

### Primers and probes

The TaqMan primers and probes were designed using Primer Express 3.0 (Applied Biosystems) and all other primers were designed using Primer3 [30]. All primers were designed using sequence information from assembly WASHUC2 (may 2006), available on the Ensembl website [31].

### Molecular analysis of the K locus

For the 15 STS markers (STS_0 to STS_14), the criteria for primer design were as follows: amplicons of 100 to 250 bp, primer melting temperature ranging from 58°C to 62°C, primer length ranging from 19 to 22 bp, and primer G/C content ranging from 40% to 60%. Slope values were calculated using software from Applied Biosystems (SDS1.2) and an input of 50, 5, 0.5, and 0.05 ng (10^2 ^– 10^-2^) DNA was used in duplicate. The slope values of all markers were within the range of -3.32 ± 0.25 [16] and the R^2 ^of all markers was above 0.994. Marker STS_0, designed in the glyceraldehyde-3-phosphate dehydrogenase gene, was used to normalize the data. The qPCR experiment was performed with the Real-time PCR 7500 from Applied Biosystems. Each 25 μl qPCR reaction was comprised of 12.5 μl IQ SYBR GREEN mastermix (Biorad), 300 nM of each primer, and 20 ng of genomic DNA. Genomic DNA from two EF (one Broiler and one White Leghorn) and two LF animals (one Broiler and one White Leghorn) were tested once for all markers. The PCR program was 50°C for 2 min, a 10 min denaturation at 95°C, then 40 cycles of 95°C for 15 sec and combined annealing and extension at 60°C for 60 sec. At the end, a dissociation step was included to confirm the specificity of the product. Results were expressed in the number of cycles (Ct value) at a threshold of 100,000 ΔRn. The method described by Sijben et al. [32] was used to normalize the Ct values (KCt). All data was normalized against the Ct values of marker STS_0. Slope values were included in the calculations.

For all markers, the average KCt was calculated for both EF animals and substracted from the KCt of each LF animal (ΔKCt). When the ΔKCt of a marker was less than 0.35, no duplication was observed; when ΔKCt was between 0.35 and 0.65, the result was ambiguous and no conclusion could be given; and when ΔKCt was more than 0.65, it indicated a gain of one copy and, therefore, a duplicated marker [16].

In order to obtain the exact breakpoint, and to identify specific SNPs in this region, the PCR reaction was performed on one EF male and one LF male from two breeds (Broiler and White Leghorn). The PTC-100 Thermal Controller (MJ Research, Inc) was used. The PCR reaction (10 μl total volume) was comprised of 5 μl ABgene PCR mastermix, 400 nM of each primer, and 20 ng of genomic DNA. The PCR program was 95°C for 5 min, followed by 36 cycles of 95°C for 30 sec, 60°C for 45 sec, and 72°C for 1 min 30 sec, with a final extension at 72°C for 10 min. Amplified products were separated at 115 V for 45 min on a 1.5% agarose gel. The products of marker STS_Junction, STS_5Block, and STS_3Block were amplified and sequenced using the Applied Biosystems 3730 DNA analyzer. The standard protocol of the Big Dye Terminator Cycle Sequencing Kit v3.1 (ABI) was used. Sequence data was analyzed using Pregap4 and Gap4 of the Staden Software Package [33]. The Pregap4 modules were used to prepare the sequence data for assembly (quality analysis). Gap4 was used for the final sequence assembly of the Pregap4 output files (normal shotgun assembly).

In addition, PCR reactions were performed on the breakpoint junction in twelve EF and twelve LF animals using the breakpoint junction marker STS_break (Table 1). Eight different lines were used: four EF and four LF lines consisting of four Broiler, two White Leghorn, and two Brown Layer lines. From each line, three animals were used in the experiment. The three LF White Leghorn animals were female. The PCR method was similar to that described above.

### The TaqMan K test

**Figure 3:**
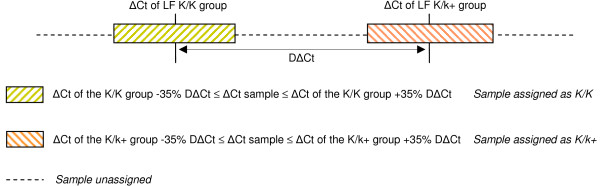
Range of ΔCt used to identify the genotype of the tested animals.

Standard curves were generated using the SDS1.2 software from Applied Biosystems with a DNA concentration of 5, 0.5, and 0.05 ng in triplicate. Marker STS_control had a R^2 ^value of 0.995 and a slope of -3.36. Marker STS_break had a R^2 ^of 0.977 and a slope of -4.31. For marker STS_break, no marker could be developed with a higher R^2 ^or a higher slope. Each 25 μl qPCR reaction was comprised of 12.5 μl ABgene PCR master mix, 300 nM of each primer, 100 nM of each probe, and 5 ng genomic DNA. The breakpoint junction and control primers and probes were used in multiplex within one reaction. The experiments were performed using the same PCR program used in the qPCR experiments, but without a dissociation step. Based on the results, the threshold was kept at 9200 ΔRn for all calculations. The difference in the number of cycles between the breakpoint junction and control marker was calculated (ΔCt = Ct FAM - Ct VIC). The difference between the average ΔCt of eight reference animals (four K/K and four K/k+) was used to calculate the DΔCt (DΔCt = ΔCt K/K - ΔCt K/k). This DΔCt was then used to calculate a range of ΔCt values to distinguish between K/K and K/k+ (Figure 3). An animal was assigned as homozygous (K/K) if the ΔCt was in the range of -35% to +35% DΔCt of the average from the homozygous reference animals. An animal was assigned as heterozygous (K/k+) if the ΔCt was in the range of -35% or +35% DΔCt of the average from the heterozygous reference animals. The ΔCt values outside these ranges were considered to be unassigned and when a tested animal was placed into the wrong genotype it was considered to be incorrectly assigned (false positive).

## List of abbreviations

bp: basepair; BL: Brown Layer; BR: Broiler; cM: centi Morgan; Ct: Cyclesneeded to reach Threshold; DΔCt: the difference between the ΔCt of K/Kand K/k+; EF: early feathering; ev21: endogenous virus 21; kb: kilobasepairs; LF: late feathering; PRL: prolactin; PRLR: prolactin receptor; qPCR: quantitative PCR; STS: sequence-tagged site; WL: White Leghorn; ΔKCt: difference in corrected C_t _of a marker between the average of the control samples and an affected sample; ΔCt: difference in uncorrected C_t _of a marker between the average of the control samples and an affected sample or the difference between the Ct value of the breakpoint marker and the control marker.

## Authors' contributions

MGE and AAAV drafted the manuscript and designed, conducted, and analyzed the experiments. APJ, RPMAC, and MAMG participated in the design of the experiments and helped substantially with manuscript preparation and editing. All authors read and approved the final manuscript.
